# 1036. Reporting of SARS-CoV-2 testing results among PEPFAR and non-PEPFAR countries in Sub-Saharan Africa during the first year of the COVID-19 pandemic

**DOI:** 10.1093/ofid/ofad500.066

**Published:** 2023-11-27

**Authors:** Benjamin C Johnson, Charles B Holmes

**Affiliations:** Johns Hopkins School of Medicine, Baltimore, MD; Georgetown University, Washington, District of Columbia

## Abstract

**Background:**

The U.S. President’s Emergency Plan for AIDS Relief (PEPFAR) is the largest global health program dedicated to addressing a single disease. In its first ten years, PEPFAR invested one-fifth of country budgets on governance and health systems strengthening, including laboratories, surveillance networks, and long-term training of health care workers. In addition to furthering the HIV response and promoting the sustainability of health programs, it has been argued these investments built capacity to respond to novel public health threats such as SARS-CoV-2.

**Methods:**

Testing data from 1/1/2020 through 2/17/2021 were downloaded from *Our World in Data*, an online database that collates information from national and international health agencies. These data were cross-referenced against publicly reported testing totals from Africa CDC and the World Health Organization. The data set did not include testing reported by governments through non-machine-readable formats such as social media or press releases. PEPFAR data were drawn from PEPFAR.gov.

**Results:**

Of the 18 countries in sub-Saharan Africa (SSA) that reported testing data one year into the pandemic, 16 (88.9%) were PEPFAR countries (Table 1). Among PEPFAR countries in SSA, 16 of 27 countries (59.3%) reported SARS-CoV-2 tests performed, compared with 2 of 21 non-PEPFAR countries (9.5%, OR = 13.8). Among countries that reported testing, PEPFAR countries had a per-capita testing rate nearly 3 times that of non-PEPFAR countries (28.7 tests per 1,000 people vs. 9.6 tests per 1,000 people), suggesting greater testing capacity. PEPFAR countries reported higher COVID-19 case rates, likely due to increased testing, however this could also be due to regional differences in incidence (Table 2). Mean GDP per capita was 62% higher in non-PEPFAR SSA countries, making it unlikely that financial resources alone account for the observed differences.

**Figure 1.**
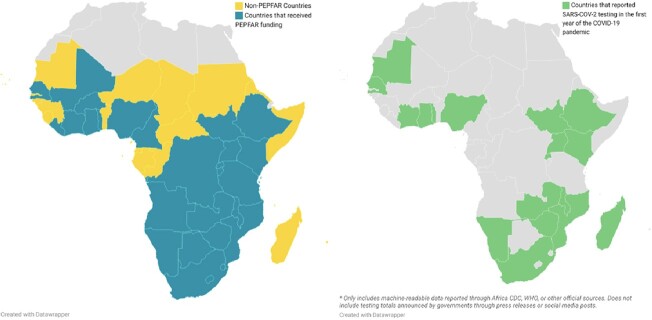
Countries that have received PEPFAR funding and countries that reported SARS-CoV-2 testing totals during the first year of the coronavirus pandemic

**Table 1.**
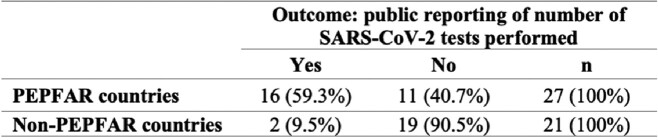
Reporting of SARS-CoV-2 testing in sub-Saharan Africa among PEPFAR and non-PEPFAR countries 1 year into the COVID-19 pandemic

**Table 2.**
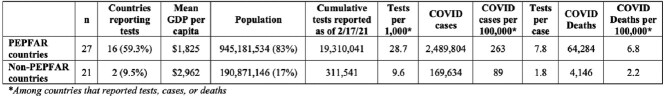
COVID-19 indicators and characteristics of PEPFAR and non-PEPFAR countries in sub-Saharan Africa

**Conclusion:**

PEPFAR countries in SSA were more likely than non-PEPFAR countries to report SARS-CoV-2 testing during the first year of the COVID-19 pandemic and also reported higher per-capita testing rates. This further supports anecdotal evidence that PEPFAR’s historical investments in health systems strengthened local capacity to respond to non-HIV public health emergencies such as COVID-19.

**Disclosures:**

**All Authors**: No reported disclosures

